# Analysis of Antidepressants Utilization for Patients Visiting Psychiatric Out-Patient Clinic in a Tertiary Care Hospital

**DOI:** 10.3390/healthcare10102081

**Published:** 2022-10-19

**Authors:** Seema Mehdi, Kishor Manohar, Atiqulla Shariff, Shahid Ud Din Wani, Mansour Almuqbil, Sultan Alshehri, Faiyaz Shakeel, Mohammad T. Imam, Kamsagara L. Krishna

**Affiliations:** 1 Department of Pharmacology, JSS College of Pharmacy, JSS Academy of Higher Education & Research, Mysuru 570015, India; 2 Department of Psychiatry, JSS Medical College and Hospital, JSS Academy of Higher Education & Research, Mysuru 570015, India; 3 Department of Pharmacy Practice, JSS College of Pharmacy, JSS Academy of Higher Education & Research, Mysuru 570015, India; 4 Department of Pharmaceutical Sciences, School of Applied Science and Technology, University of Kashmir, Srinagar 190006, India; 5 Department of Clinical Pharmacy, College of Pharmacy, King Saud University, Riyadh 11451, Saudi Arabia; 6 Department of Pharmaceutics, College of Pharmacy, King Saud University, Riyadh 11451, Saudi Arabia; 7 Department of Clinical Pharmacy, College of Pharmacy, Prince Sattam Bin Abdulaziz University, Al-Kharj 11942, Saudi Arabia

**Keywords:** antidepressants, depression, drug utilization, mental health, prescription pattern

## Abstract

Depression is a prevalent mental health condition treated with antidepressants and other psychotropic medications. This study aimed to assess the utilization pattern of antidepressants among patients visiting the outpatient clinic of the psychiatry department of a tertiary care hospital. The study included the patients who visited the study site and fulfilled the mental and behavioral diagnostic criteria for depression. The demographic and clinical details, including drugs prescribed, were documented in a study-specific data collection form. The ratio of Prescribed Daily Dose to Defined Daily Dose (PDD: DDD) was calculated to assess the adequacy of antidepressant utilization. Data total of 154 patients were collected. A total of 22 psychotropic drugs were used among the study patients as mono (*n* = 70), dual (*n* = 69), triple (*n* = 10), or quadruple therapy (*n* = 1). Escitalopram was the most often prescribed antidepressant out of the nine antidepressants alone and in combination and was used in slightly high doses (PDD: DDD ratio 1.6). Sertraline, paroxetine, and desvenlafaxine, were used in adequate doses (PDD: DDD between 1 and 1.1), and fluoxetine, duloxetine, amitriptyline, imipramine, and mirtazapine, were used in inadequate doses (PDD: DDD <0.5). Our study findings reveal the need for continuous assessment of antidepressants medications usage in a hospital set up.

## 1. Introduction

Depression is a mental health condition characterized by melancholy, loneliness, misery, and low self-esteem [1]. It may be manifested as sad or low feelings with distrust and fear, poor concentration, decreased attention in routine tasks, mental lethargy, insomnia or increased sleep, severe weight fluctuations, altered diet patterns, psychomotor agitation or retardation, a sense of guilt or insignificance, and decreased energy or libido lasting for at least two weeks [2,3].

Depression and anxiety disorders are the most prevalent mental health conditions, impacting more than 15% of people at certain phase during their lives [3]. A suicide attempt is made by 10–15% of those with severe depression. More than 60% of persons with depression do not pursue medical advice because of the stigma attached to mental health disorders and their negative impact on their personal and professional lives [2]. Therefore, it is critical that depression symptoms are identified and promptly addressed.

Monoamine oxidase inhibitors (MAOIs), serotonin and norepinephrine reuptake inhibitors (SNRIs), tricyclic antidepressants (TCAs), selective serotonin reuptake inhibitors (SSRIs), and atypical antidepressants, are the five main categories of antidepressants specifically utilized in psychopharmacotherapy [4]. Sertraline (SSRIs), duloxetine (SNRIs), mirtazapine (atypical antidepressant), and escitalopram (SSRIs), constituted the largest percentage (96.36%) of antidepressant prescriptions. Depending on the symptoms, past medical history, and co-occurring psychiatric illnesses, different drug combinations (antidepressant polypharmacy) are used to manage depression effectively; antidepressants are also widely prescribed in patients following screening in primary care and a prolonged use of antidepressants are found in older patients [5,6,7,8,9].

Only a few studies have examined the antidepressant prescription trends in India. Given the increasing usage of antidepressants and limited evidence of their long-term efficacy and safety, it is of utmost significance to study the real-world prescribing trends that can offer valuable guidance to optimize the use of antidepressants for achieving the desired clinical outcomes while keeping cost-effectiveness. Therefore, this study aimed to assess drug utilization patterns to facilitate the rational use of the antidepressant in patients with depressive disorders.

## 2. Materials and Methods

### 2.1. Method

A single-centered observational study was carried out at the psychiatry outpatient unit of JSS Hospital, Mysuru, Karnataka, India. The study involved 154 patients and was conducted for 3 months. We used the following inclusion/exclusion criteria for the study:

#### 2.1.1. Inclusion Criteria

Patients above 18 years of age, diagnosed with depression per the mental and behavioral diagnostic criteria of the International Classification of Disease (ICD-10) criteria, and ready to give consent were included in the study.

#### 2.1.2. Exclusion Criteria

Patients who were not in a state of giving consent and patients with terminal comorbidities, cognitive or hearing impairment, or any other medical condition which is likely to influence participation in the study were excluded from the study.

The prescriptions and case records of patients satisfying inclusion criteria were reviewed upon approval by the institutional Human Ethics Committee (JSSCPM/IHEC/2019/016). The patients were classified based on the severity of depression as per the PHQ9 questionnaire [10]. The patients’ demographic details were reviewed, including gender, education level, occupation, monthly income, socioeconomic class, marital status, social habits, residence, and dietary habits. The socio-economic status of study participants was analyzed following modified Kuppusawamy scales [11]. The prescriptions were assessed for parameters such as the number of patients utilizing each drug and distribution of the same based on disease severity. The collected data were entered into a data collection form designed explicitly for the study. All the required information was obtained through the practitioners treating the patients, and there was no direct interaction with the patients involved.

The Defined Daily Dose (DDD) is the assumed average maintenance dose per day for a drug used for its main indication in adults, as taken from WHOCC_ATC/DDD index. Prescribed Daily Dose (PDD) for each drug was calculated as follows [11]:

Prescribed Daily Dose = Total amount of drug prescribed/Duration of prescription

Finally, the ratio of PDD to DDD was calculated.

### 2.2. Statistical Analysis

The collected data were analyzed using the Statistical Package for the Social Sciences (SPSS) version 24.0, IBM Armonk, New York, NY, USA. The categorical data were reported as percentages, while the continuous data were reported as mean ±SD. The Chi-square test was used to examine the association between the dependent and independent categorical variables. A probability value of <0.05 was considered statistically significant, and any value less than 0.01 was considered highly significant.

## 3. Results

The study included a total of 154 patients. The majority of the study population included females (105, 68.2%). The mean (±SD) age of the patients was 39.5 (±12) years. A majority of the patients were illiterates (44, 28.5%), unemployed (120, 78%), married (126, 81.8%), living in rural areas (97, 63%), and belonged to the lower middle class (31, 20.1%). The detailed demographic details of the study patients are elucidated in Table 1.

**Figure 1:**
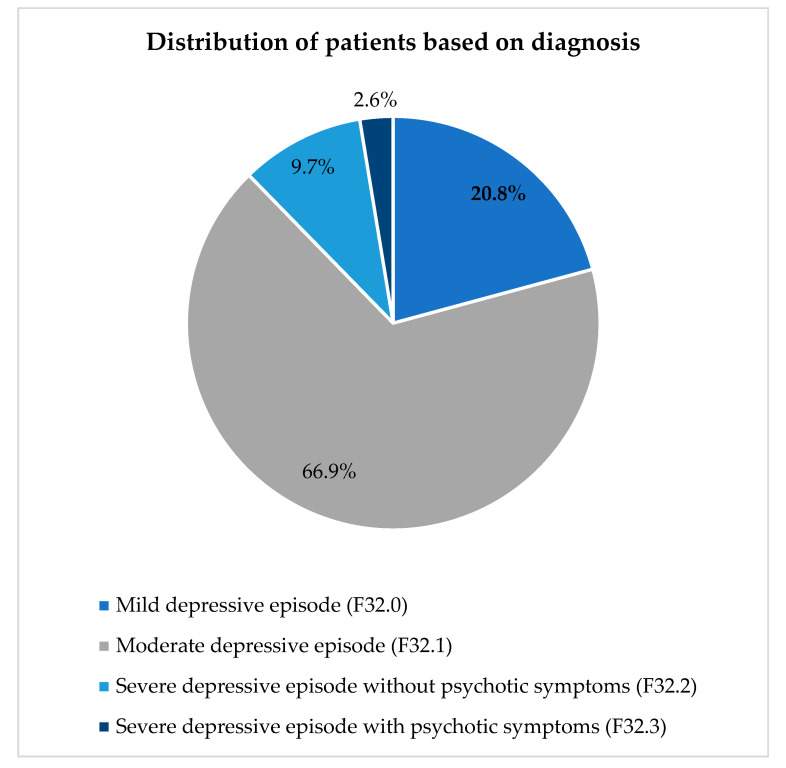
Distribution of patients based on the severity of depressive symptoms.

- The patients were diagnosed based on the severity of depression—the distribution is visually elucidated in Figure 1. The highest number of patients (103, 66.9%) were diagnosed with moderate depressive episodes.
- Moreover, the study revealed that 35 (22.7%) patients had other comorbid conditions, with generalized idiopathic epilepsy and epileptic syndromes (12/35, 34.2%) being the most common (Table 2).

**Table 2:** Prevalence of comorbid conditions among study participants.

Comorbid Conditions	ICD-10 Code	No. of Patients (%)
Generalized idiopathic epilepsy and epileptic syndromes	G40.3	12 (7.8)
Dyspepsia (Somatoform autonomic dysfunction)	F45.3	11 (7.1)
Primary hypertension	I10	5 (3.2)
Migraine, unspecified	G43.9	3 (1.9)
Alzheimer’s disease, unspecified	G30.9	2 (1.3)
Old myocardial infarction	I25.2	1 (0.6)
Parkinson disease	G20	1 (0.6)

A total of 22 drugs were used, among which nine were antidepressants, and 13 were other psychotropic agents. The median number of antidepressants prescribed was two (IQR: 1–2) per patient. The details of antidepressants and other psychotropic medications prescribed for the study population, along with their PDD/DDD scores and the percentage of usage of individual drugs based on diagnosis, are summarized in Table 3.

**Figure 2:**
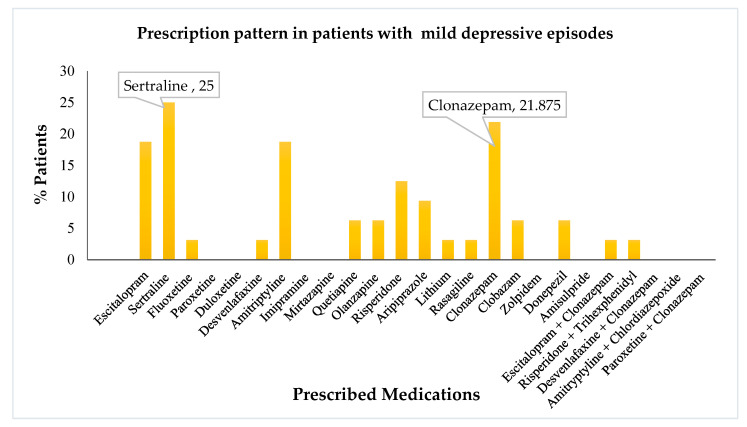
Prescription pattern of antidepressants and other psychotropics in patients with mild depressive episodes.

**Figure 3:**
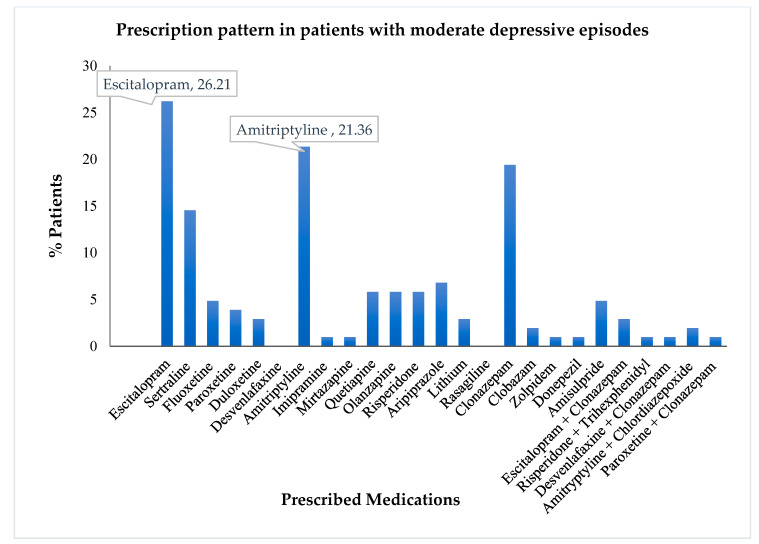
Prescription pattern of antidepressants and other psychotropics in patients with moderate depressive episodes.

- Amongst the drugs prescribed, escitalopram, amitriptyline, and clonazepam, were the most common. Further, we evaluated the distribution and utilization of various drugs based on the use percentage in different diagnoses. The drug use pattern in mild depressive patients is visually depicted in Figure 2.
- It is noteworthy that sertraline and clonazepam were the most commonly prescribed agents for mild depressive disorder. Escitalopram and amitriptyline followed in succession. Risperidone and aripiprazole were used occasionally.
- A similar analysis was performed using the drug use pattern data among moderately depressive patients. The visual representation of the same is depicted in Figure 3. Similar to the treatment pattern discussed previously, moderate depressive episodes utilized escitalopram, amitriptyline, sertraline, and clonazepam, as the most commonly prescribed drugs.

**Figure 4:**
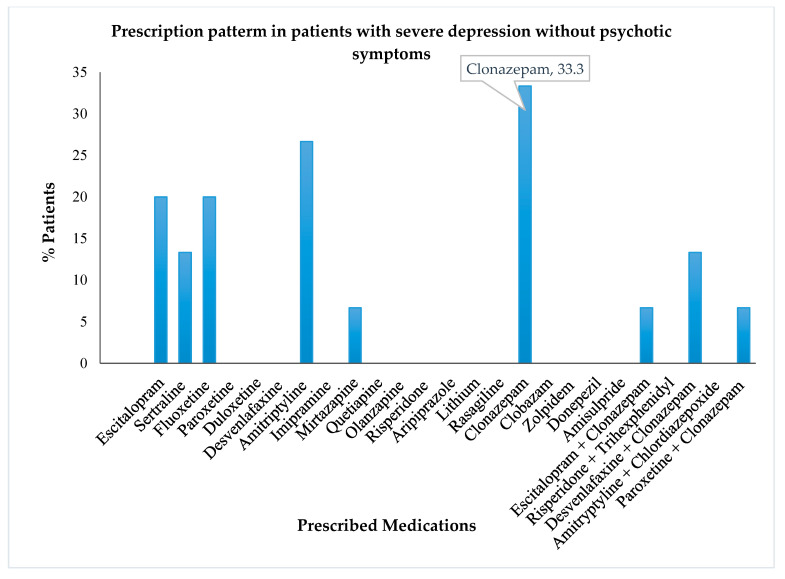
Prescription pattern of antidepressants and other psychotropics in patients with severe depressive episodes without psychotic symptoms.

**Figure 5:**
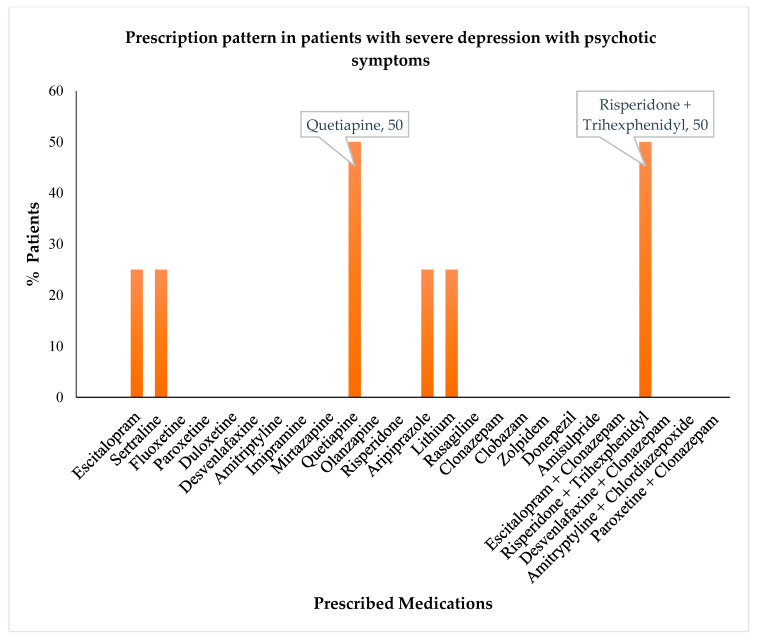
Prescription pattern of antidepressants and other psychotropics in patients with severe depression with psychotic symptoms.

The analysis of severely depressed patients without psychotic symptoms revealed that the highest numbers of these patients were treated with clonazepam followed by amitriptyline, fluoxetine, and escitalopram (Figure 4). At the same time, the severely depressed patients with psychotic symptoms were primarily treated with quetiapine or a combination of risperidone and trihexyphenidyl (Figure 5).

**Figure 6:**
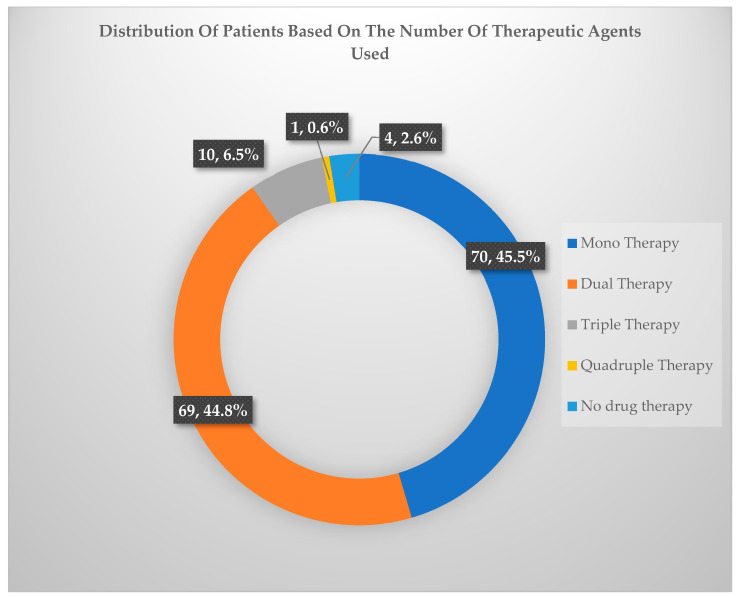
Distribution of patients based on the number of therapeutic agents used.

- In addition to studying the prescription pattern of different types of antidepressants and psychotropic drugs in patients with varying severity of depression, we also analyzed whether these patients were treated with single or multiple drug regimens (Figure 6 and Table 4).
- It was found that most patients were treated either with monotherapy or dual therapy. These patients amounted to a cumulative total of 92.67%. Only 6.67% of patients were treated with triple therapy, while an even smaller number (0.67%) were treated using the quadruple therapeutic approach.

## 4. Discussion

In the present study, more females (68.2%) than males were diagnosed with depression, which agreed with the WHO factsheet stating: “More women are affected by depression than men [12]”. The patients had a mean age of 39.5 (±12), similar to the results of a European study, which demonstrated that antidepressants were the second most frequently prescribed psychotropic drugs in the vast majority of patients aged 35 to 49 years [13]. Moreover, a higher percentage of study subjects fell into the category of people with low literacy levels, unemployed people, or those who belonged to upper lower socioeconomic class (IV). The results agreed with a recent mental health study that concluded that higher educational attainment mediates socioeconomic position and is linked to a lower incidence of depression at age 40, and other studies that illustrated that low socioeconomic status is associated with increasing cases of depression [14,15,16]. Additionally, in our study, a higher percentage of depressive patients were married. However, not many studies establish a relationship between marital status and depression and need extensive review and study. Our study did not reflect any impact of bad social habits on depression. Furthermore, a higher percentage of the study population belonged to the rural class, which might be attributed to the fact that 1/3rd of the Indian population resides in rural areas and is associated with factors such slow education, poverty, and environmental factor adversities. Finally, a higher proportion of the subjects were nonvegetarian; the observation conflicted with the published studies, which conclude that vegetarian diets are associated with a higher risk of depression [17].

The patients visiting the psychiatric unit were categorized as suffering from mild, moderate, and severe, depressive episodes. Among them, the prevalence of moderate depressive episodes was the highest (*n* = 103; 66.9%). The antidepressant was chosen based on the severity of the disease, efficacy of the drug, comorbid conditions, and patient tolerance. Escitalopram, a selective serotonin reuptake inhibitor (SSRI), was the most widely prescribed antidepressant, followed by amitriptyline and clonazepam, belonging to the class of tricyclic antidepressants and benzodiazepine derivatives, respectively. Our findings were similar to previous research findings that elucidated the preferential prescribing pattern of escitalopram and illustrated that prescribing escitalopram to patients suffering from major depressive disorder significantly reduced the occurrence of a relapse [18]. Another meta-analysis concluded that when evaluated for efficacy based on the Montgomery-Asberg Depression Rating Scale and the Hamilton Depression Rating Scale, escitalopram was nearly equal to or superior to other SSRIs and serotonin-noradrenaline reuptake inhibitors, and prevented the recurrence of major depressive disorder [19].

Sertraline was prescribed most for mild depressive disorders. This drug choice is supported by a clinical trial that established the drug efficacy and superiority in primary-care patients with milder forms of depression [20].

The most common antidepressant for moderate depression was escitalopram (26.21%), which was in agreement with the Practice Guideline for the Treatment of Patients with depression that recognize SSRIs as the first-choice agents for therapeutic management of depressive disorders [21,22]. The prescription pattern also revealed that moderately severe patients with no psychotic symptoms were prescribed clonazepam.

Since studies have shown that clonazepam has the potential to enhance the effects of SSRIs and can partially inhibit their negative effects, it is the preferred benzodiazepine when used with antidepressants. In our analysis, benzodiazepines were still frequently prescribed, but perhaps at a lower rate than in earlier Indian studies that looked at prescription trends in patients with depressive disorders [23,24]. The benzodiazepines were used in previous studies regarding high comorbid anxiety in depression and the expected exacerbation of anxiety with antidepressants. According to the National Institute for Health and Clinical Excellence (NICE) recommendations, benzodiazepines may be beneficial for up to two weeks early in the course of treatment, especially when combined with SSRIs, and use beyond this timeline is discouraged as these drugs may cause addiction. Many patients may use benzodiazepines for years with no recognized benefits, but harmful side effects. Hence, prescribers must advise benzodiazepines in the smallest dose and for the shortest possible time [25].

The treatment pattern of patients with psychotic symptoms differed from those without psychotic symptoms and included quetiapine, and a fixed dose combination of risperidone + trihexyphenidyl, as the frequently prescribed drugs. This was in conjunction with a study that illustrated that treatment with risperidone effectively alleviates psychotic depression, and the influence of risperidone on dopaminergic activity is associated with its efficacy [26]. Another study showed a similar drug combination therapy pattern to help alleviate psychotic symptoms [27].

Furthermore, in our study, a significantly higher percentage of patients were advised monotherapy or dual therapy. In monotherapy patients, the most frequently prescribed antidepressants were amitriptyline and escitalopram. Coincidently, in the patients on dual therapy, the most frequently prescribed combination was escitalopram and amitriptyline. Very few patients were treated with more than two drugs. The additional medicines were added based on the severity of the disease.

Comparing DDD with its PDD helps evaluate and interpret drug utilization figures. The defined daily dose (DDD) is the assumed average maintenance dose per day for a drug used for its main indication in adults [28]. Conversely, the prescribed daily dose (PDD) is the average dose prescribed according to a representative sample of prescriptions. The ratio of PDD to DDD is often used to indicate the adequacy of dosing. A ratio of less than 1, as was mainly seen in the case of clonazepam, indicates under-dosing. A high PDD/DDD ratio of 1.6 was seen with escitalopram, indicating overdosing. Many other antidepressants showed a PDD to DDD ratio, which was below 1, thus reflecting the inadequacy of the dosing in these cases [29]. The reason for lower PDD could be attributed to anticipated side effects of the drugs, severity of disease, body weight, or the prescribing culture of the health provider.

## 5. Conclusions

Studying drug utilization offers an insight into changing trends in the prescription pattern. The increasing incidences of depressive illness indicate a surge in the prescription of antidepressants. Our study revealed that escitalopram was the most prescribed antidepressant alone and in combination. Polypharmacy in concomitant use of two antidepressants was practiced as frequently as monotherapy. Our findings can be further utilized for further designing studies to compare changes in the prescription pattern of antidepressant drugs.

## Figures and Tables

**Table 1:** Demographic details of the study participants.

Demographics	No. of Patients (%)
Gender	
Male	49 (31.8)
Female	105 (68.2)
Education	
Illiterate	44 (28.5)
High school certificate	43 (28)
Intermediate/diploma	20 (13)
Graduate	22 (14.3)
Primary school certificate	17 (11)
Middle school certificate	8 (5.2)
Occupation	
Professionals	7 (4.5)
Clerks	1 (0.6)
Skilled Workers and Shop & Market Sales Workers	11 (7.1)
Skilled Agricultural & Fishery Workers	9 (5.8)
Plant & Machine Operators and Assemblers	2 (1.3)
Elementary Occupation	4 (2.6)
Unemployed	120 (78)
Monthly income (INR)	
46,129–61,662	8 (5.2)
30,831–46,128	79 (51.3)
18,497–30,830	45 (29.2)
6175–18,496	22 (14.3)
Socioeconomic class	
Upper Middle (II)	7 (4.5)
Lower Middle (III)	31 (20.1)
Upper Lower (IV)	98 (63.6)
Lower (V)	18 (11.7)
Marital status	
Single	23 (14.9)
Married	126 (81.8)
Widow	5 (3.2)
Social habits	
Alcohol use	8 (5.2)
Tobacco use	13 (8.4)
Alcohol and tobacco use	10 (6.5)
None	123 (79.8)
Residence	
Rural	97 (63)
Urban	57 (37)
Diet habits	
Non-vegetarian	113 (73.3)
Vegetarian	41 (26.6)

**Table 3:** Details of antidepressants and other psychotropic drugs prescribed.

Drug Name	No. of Patients (%)	ATC Code	PDD (mg)	DDD (mg)	PDD/DDD	Mild Depression [*n* = 32 (%)]	Moderate Depression [*n* = 103 (%)]	Moderately Severe [*n* = 15 (%)]	Moderately Severe with Psychotic Symptoms [*n* = 4 (%)]
Selective serotonin reuptake inhibitors (SSRIs)									
Escitalopram	37	N06AB10	16	10	1.6	6 (18.75)	27 (26.21)	3 (20)	1 (25)
Sertraline	26	N06AB06	57	50	1.1	8 (25)	15 (14.56)	2 (13.33)	1 (25)
Fluoxetine	9	N06AB03	9.7	20	0.5	1 (3.125)	5 (4.85)	3 (20)	0
Paroxetine	4	N06AB05	22	20	1.1	0	4 (3.88)	0	0
Serotonin and norepinephrine reuptake inhibitors (SNRIs)									
Duloxetine	3	N06AX21	33	60	0.5	0	3 (2.91)	0	0
Desvenlafaxine	1	N06AX23	50	50	1	1 (3.125)	0	0	0
Tricyclic antidepressants									
Amitriptyline	33	N06AA09	20	75	0.26	6 (18.75)	22 (21.36)	4 (26.67)	0
Imipramine	1	N06AA02	25	100	0.25	0	1 (0.97)	0	0
Serotonin and α-2 adrenergic antagonists									
Mirtazapine	2	N06AX11	7.5	30	0.25	0	1 (0.97)	1 (6.67)	0
Diazepines, oxazepines, thiazepines and oxepines									
Quetiapine	10	N05AH04	108	400	0.27	2 (6.25)	6 (5.83)	0	2 (50)
Olanzapine	8	N05AH03	8.75	10	0.87	2 (6.25)	6 (5.83)	0	0
Other antipsychotics									
Risperidone	15	N05AX08	1.63	5	0.32	4 (12.5)	6 (5.83)	0	0
Aripiprazole	11	N05AX12	8.45	15	0.56	3 (9.375)	7 (6.80)	0	1 (25)
Lithium	5	N05AN01	482.6	1773.3	0.27	1 (3.125)	3 (2.91)	0	1 (25)
Monoamine oxidase B inhibitors									
Rasagiline	1	N04BD02	1	1	1	1 (3.125)	0	0	0
Benzodiazepine derivative									
Clonazepam	31	N03AE01	1.9	8	0.23	7 (21.875)	20 (19.42)	5 (33.33)	0
Clobazam	4	N05BA09	7.5	20	0.37	2 (6.25)	2 (1.94)	0	0
Zolpidem	1	N05CF02	5	10	0.5	0	1 (0.97)	0	0
Anticholinergics									
Donepezil	3	N06DA02	5	7.5	0.66	2 (6.25)	1 (0.97)	0	0
Benzamides									
Amisulpride	5	N05AL05	160	400	0.4	0	5 (4.85)	0	0
Fixed dose combination therapy									
Escitalopram + Clonazepam	5	Not Applicable				1 (3.125)	3 (2.91)	1 (6.67)	0
Risperidone + Trihexyphenidyl	4	Not Applicable				1 (3.125)	1 (0.97)	0	2 (50)
Desvenlafaxine + Clonazepam	3	Not Applicable				0	1 (0.97)	2 (13.33)	0
Amitryptyline + Chlordiazepoxide	2	Not Applicable				0	2 (1.94)	0	0
Paroxetine + Clonazepam	2	Not Applicable				0	1 (0.97)	1 (6.67)	0

**Table 4:** Details of mono, dual, triple, or quadruple therapy prescribed.

Type of Drug Therapy	Drug Name	No. of Patients [*n* = 150 (%)]
Monotherapy		70 (46.6)
One antidepressant (*n* = 40, 26.6%)	Amitriptyline	14 (9.3)
	Escitalopram	13 (8.6)
	Sertraline	6 (4)
	Fluoxetine	4 (2.6)
	Desvenlafaxine	1 (0.6)
	Imipramine	1 (0.6)
	Mirtazapine	1 (0.6)
One psychotropic drug (*n* = 30, 20%)	Clonazepam	8 (5.3)
	Risperidone	8 (5.3)
	Quetiapine	6 (4)
	Aripiprazole	3 (2)
	Olanzapine	2 (1.3)
	Amisulpride	2 (1.3)
	Lithium	1 (0.6)
Dual therapy		69 (46)
Two antidepressants (*n* = 13, 8.6%)	Escitalopram and amitriptyline	9 (6)
	Sertraline and amitriptyline	2 (1.3)
	Escitalopram and mirtazapine	1 (0.6)
	Paroxetine and amitriptyline	1 (0.6)
Two psychotropic drugs (*n* = 7, 4.6%)	Clonazepam and risperidone	1 (0.6)
	Rasagiline and donepezil	1 (0.6)
	Quetiapine and aripiprazole	1 (0.6)
	Amisulpride and aripiprazole	1 (0.6)
	Olanzapine and lithium	1 (0.6)
	Olazapine and clobazam	1 (0.6)
	Clobazam and donepezil	1 (0.6)
One antidepressant and one psychotropic drug (*n* = 39, 26%)	Sertraline and clonazepam	8 (5.3)
	Escitalopram and clonazepam	6 (4)
	Escitalopram and aripiprazole	4 (2.6)
	Sertraline and risperidone	4 (2.6)
	Fluoxetine and clonazepam	2 (1.3)
	Duloxetine and quetiapine	2 (1.3)
	Escitalopram and lithium	2 (1.3)
	Amitriptyline and clonazepam	1 (0.6)
	Duloxetine and clonazepam	1 (0.6)
	Paroxetine and aripiprazole	1 (0.6)
	Sertraline and amisulpride	1 (0.6)
	Paroxetine and amisulpride	1 (0.6)
	Fluoxetine and zolpidem	1 (0.6)
	Escitalopram and olanzapine	1 (0.6)
	Sertraline and olanzapine	1 (0.6)
	Amitriptyline and olanzapine	1 (0.6)
	Escitalopram and clobazam	1 (0.6)
	Amitriptyline and clobazam	1 (0.6)
Combination therapy (*n* = 10, 6.6%)	Desvenalafaxine + clonazepam	3 (2)
	Escitalopram + clonazepam	3 (2)
	Risperidone + trihexyphenidyl	2 (1.3)
	Amitriptyline + chlordiazepoxide	2 (1.3)
Triple therapy		10 (6.6)
Two antidepressants and one psychotropic drug (*n* = 1, 0.6%)	Sertraline, fluoxetine and clonazepam	1 (0.6)
One antidepressant and two psychotropic drug (*n* = 4, 2.6%)	Fluoxetine, donepezil and risperidone	1 (0.6)
	Sertraline, clonazepam and risperidone	1 (0.6)
	Sertraline, clonazepam and olanzapine	1 (0.6)
	Paroxetine, clonazepam and aripiprazole	1 (0.6)
One combination therapy and one antidepressant (*n* = 5, 3.3%)	Escitalopram + clonazepam and amitriptyline	2 (1.3)
	Paroxetine + clonazepam and amitriptyline	1 (0.6)
	Paroxetine + clonazepam and amitriptyline	1 (0.6)
	Risperidone + trihexyphenidyl and sertraline	1 (0.6)
Quadruple therapy (*n* = 1, 0.6%)	Risperidone + trihexyphenidyl, quetiapine, and lithium	1 (0.6)

## Data Availability

Not applicable.
